# Association of wrist and forearm range of motion measures with self-reported functional scores amongst patients with distal radius fractures: a longitudinal study

**DOI:** 10.1186/s12891-018-2065-z

**Published:** 2018-05-11

**Authors:** Zixian Yang, Peggy Poh Hoon Lim, Sing Hwee Teo, Huiwen Chen, Huaying Qiu, Yong Hao Pua

**Affiliations:** 1 Department of Occupational Therapy, Singapore General Hospital, Singapore, Singapore; 2 Department of Occupational Therapy, Tan Tock Seng Hospital, Singapore, Singapore; 3 Department of Occupational Therapy, Changi General Hospital, Singapore, Singapore; 4 Department of Occupational Therapy, Ng Teng Fong General Hospital, Singapore, Singapore; 5 Department of Physiotherapy, Singapore General Hospital, Singapore, Singapore

**Keywords:** Rehabilitation, Occupational therapy, Distal radius fractures, Range of motion, Function

## Abstract

**Background:**

Patients with distal radius fractures (DRF) often have limited range-of-motion (ROM) in multiple planes of movement. No studies have comprehensively examined the impact of various ROM limitations on physical function.

**Methods:**

We performed a multi-center, longitudinal study of 138 patients with conservatively managed DRF. ROM measures were taken at initial evaluation, and at 4 and 8 weeks later. Self-reported physical function was indexed by the Quick Disabilities of the Arm, Shoulder and Hand (QuickDASH).

**Results:**

Wrist extension, active thumb opposition and a full composite grip were amongst the strongest ROM measures associated with functional scores over time. However, wrist radial deviation and forearm pronation were non-significantly associated with functional scores.

**Conclusion:**

Given that ROM is potentially modifiable, the identification of important ROM measures associated with QuickDASH scores can potentially facilitate patient education and refine interventions to optimize functional recovery. Well-designed randomized intervention studies are however needed to confirm these association findings.

## Background

Distal radius fractures (DRFs) are a common occurrence in clinical practice and account for 20% of all fractures seen in emergency departments [1, 2]. Due to the involvement of the wrist joint, patients often have limited range of motion (ROM) in multiple planes of movement - namely, wrist flexion and extension, wrist radial and ulnar deviation, forearm supination and pronation. As the wrist joint is imperative for proper function of the hand, fundamental hand functions such as making a full composite grip and thumb opposition are also often affected [3] despite not being injured. These can adversely impact on one’s ability to perform activities of daily living (ADLs), work or leisure, which causes loss of productive work hours, school in-attendance, loss of independence, and even lasting disability – extending beyond the direct healthcare costs [4–6]. Hence, occupational therapists and patients spend considerable amount of time addressing these multiple ROM impairments with the aim of functional restoration.

To our knowledge, the published literature on the relationship between the various ROM measures and physical function amongst patients with conservatively managed DRFs is limited to 2 small cross-sectional studies [7, 8]. Kilic et al. (*n* = 29) examined ROM measures as a total (combined) arc-of-motion in the planes of wrist flexion/extension and forearm supination/pronation and thus, could not tease apart the isolated associations of the specific ROM measures with physical function [7]. The other study by Tremayne et al. (*n* = 20) examined only one ROM measure (wrist extension) [8]. Although several longitudinal studies have described the ROM measures and functional scores over time [9–11], these studies were largely descriptive and did not formally examine the associations between the specific ROM measures and physical function using adequately controlled multivariable analyses. Thus, we believe that a consistent and comprehensive examination of the ROM-physical function association in patients with conservatively managed DRFs has not been previously presented.

As ROM is potentially modifiable, the identification of important ROM measures associated functional scores can facilitate patient education and help refine rehabilitation interventions to optimize functional outcomes. Thus, the aim of this study was to examine the longitudinal associations of 8 forearm, wrist and hand ROM measures with self-reported functional scores amongst patients with non-complicated, conservatively managed DRFs. Specifically, the ROM measures include wrist flexion and extension; wrist radial and ulnar deviation; forearm supination and pronation; active thumb opposition and the ability to make a full composite grip.

## Methods

### Design

This study was a retrospective, longitudinal, multi-center study involving four major outpatient occupational therapy hand clinics in Singapore. Patients were evaluated at three time points. Specifically, the baseline time point was taken to be at the commencement of active mobilization, and the subsequent two time points were set at the commencement of passive and strengthening exercises, respectively.

### Participants and setting

All patients with conservatively managed, unilateral DRFs who were referred to four outpatient occupational therapy hand clinics during the period of April to June 2015 were included in the study. Patients were excluded if there were other upper limb fractures, bilateral injuries, or associated complicating injuries such as tendon, ligament or nerve injuries. Patients who had been treated by other institutions as well as those who developed complex regional pain syndrome (CRPS) subsequently were also excluded. The study received ethics approval from all participating institutions. Demographic and clinical information such as age, gender, injured side, handedness (the preferred hand for performing certain unimanual tasks [12]), edema, time from injury to first therapy session were collected from therapy records of the patients involved in the study. Of note, edema was measured with a soft measuring tape, using a figure-of-eight method [13].

### Treatment providers

Patients included in this study were treated by 34 occupational therapists across four hand clinics. There was an equal distribution of therapists who had 5 or more years of clinical experience in hand therapy (*n* = 17), and those who had less than 5 years (*n* = 17). Across the four hand clinics, the average number of years of clinical experience was 6.78 years. 8 therapists were masters/postgraduate level occupational therapists.

### Range of motion

Wrist goniometers (Smith & Nephew; Kinetec; North Coast Medical; Baseline) were used to measure active range of motion (ROM) of wrist flexion, extension, radial and ulnar deviation as well as forearm supination and pronation in the injured upper limb. Wrist flexion and extension were measured with the forearm in neutral on a stable surface, and the goniometer on the radial aspect of the wrist, aligned along the third metacarpal. Wrist radial and ulnar deviation were measured with the forearm in pronation on a flat surface and elbow slightly flexed. Goniometer measurements were taken along the line of the third metacarpal and the radius bone. Forearm supination and pronation measurements were performed with elbow flexed at 90 degrees, at the side of the body and goniometer readings aligned to the imaginary line between the radial and ulnar styloids. Active thumb opposition was recorded using the modified Kapandji score [14]. Measurements of distance from tip of finger to distal palmar crease (DPC) were performed using flat edge goniometers (Roylan; Jamar; Sammon Preston; North Coast Medical), where 0 cm indicates a full composite grip. All methods of measurement were standardized across all four hand clinics and were performed by occupational therapists at the commencement of active and passive mobilization as well as strengthening phases.

### The QuickDASH outcome measure

The QuickDASH is a shortened version of the original 30-item Disabilities of the Arm, Shoulder and Hand (DASH) questionnaire [15]. Developed to measure physical function and symptoms in people with musculoskeletal disorders of the upper limb, the QuickDASH is an 11-item self-administered questionnaire scored on a 5-point scale. The QuickDASH score ranges between 0 and 100, where a lower score indicating lower disability levels. A QuickDASH score would not be generated if more than 10% of the items were left blank [16]. Noteworthy, the QuickDASH is a well-accepted patient-reported outcome [17] and we chose a self-report measure because it is easier for patient to express their perception of the impact of their injury on function as well as making it easier for the clinician to analyse [18].

### Statistical analysis

Continuous variables were presented as means with standard deviations (SD) for normally distributed data, and medians with interquartile ranges (IQRs) for non-normally distributed data, whilst categorical variables were presented as frequencies with percentages. We modeled the time course of the QuickDASH scores using a generalized least-squares model that included all available observations from all time points, and we accounted for within-patient correlation over time using a first order autoregressive covariance structure [19, 20]. To avoid assuming linear time trends, actual assessment dates were used and time (weeks since baseline time point) was modeled flexibly as a restricted cubic spline [19, 21].

To assess the independent associations of QuickDASH with the various ROM measures, these measures were each used as a time-varying independent variable in separate models and the covariates included age, sex, edema, hand dominance, time since fracture, and treatment center. To evaluate whether the associations of the ROM measures with QuickDASH changed over time, we considered their first-order interactions with time. To facilitate interpretation and comparison of the results, we scaled the regression coefficients of the ROM measures by their IQRs such that the coefficients may be interpreted as comparing patients with relatively high (75th percentile) versus low (25th percentile) ROM on the QuickDASH scores [19]. Besides allowing valid comparisons, IQR regression coefficients represented a more clinically meaningful distinction than the conventional one-unit (1° or 1 cm) change in the ROM values.

We assessed the appropriateness of all models using residual plots and we used *R* software, version 3.2.3 (http://www.r-project.org), for all analyses.

## Results

### Sample characteristics and time course of physical measures

A total of 138 patients were included in the study. Table 1 shows the baseline characteristics of the total sample. Mean age was 59 years (SD 16) and women accounted for more than half (67%) of the sample. Nearly half of the sample injured their dominant hand and the median time since injury to the first occupational therapy session was slightly more than a month. Majority of the distal radius fractures were as a result of a low energy fall (88%).

**Table 1:** Baseline characteristics

Participants demographics (*n* = 138)	
Age in years, mean (SD)	59 (16)
Gender, male	45 (33%)
Injured dominant hand	68 (49%)
Injury due to low energy fall	121 (88%)
Weeks from injury to first occupational therapy session, median (IQR)	5.3 (4.4–7.0)
Weeks from baseline session to PROM phase, median (IQR)	4.1 (2.9–6.0)
Weeks from baseline session to strengthening phase, median (IQR)	8.0 (6.0–11.8)

Table 2 shows the mean values of all physical measures at all time points, except that of the QuickDASH scores which were expressed as medians with IQRs. Overall, there was improvement in all physical measures and QuickDASH scores from baseline to the commencement of strengthening phase.

**Table 2:** Physical measures at active, passive mobilization and strengthening phases

Physical measures	AROM^a^ (1) mean (SD)	PROM^b^ (2) mean (SD)	Strengthening (3) mean (SD)	1 vs 2	2 vs 3
Wrist flexion, degrees	28 (11)	39 (14)	44 (14)	< 0.001	< 0.001
Wrist extension, degrees	35 (15)	49 (12)	56 (11)	< 0.001	< 0.001
Wrist radial deviation, degrees	11.5 (7.5)	16.4 (7.9)	17.7 (6.6)	< 0.001	0.041
Wrist ulnar deviation, degrees	21 (8.5)	27 (10)	28.7 (9.4)	< 0.001	0.035
Forearm supination, degrees	62 (22)	78 (13)	81.5 (8.7)	< 0.001	0.039
Forearm pronation, degrees	65 (21)	75 (14)	78 (13)	< 0.001	0.041
Distance to distal palmar crease (DPC)^c^, cm	1.9 (2.1)	1.0 (1.6)	0.52 (1.06)	< 0.001	< 0.01
Active thumb opposition^d^	5.3 (2.1)	6.4 (1.9)	7.0 (1.4)	< 0.001	< 0.001
Edema, cm	1.11 (1.46)	0.78 (1.10)	0.53 (0.86)	< 0.001	0.02
QuickDASH^e^ Scores, median (IQR)	39 (24–64)	27 (16–41)	14 (5–25)	< 0.001	< 0.001

^a^Active mobilization phase

^b^Passive mobilization phase

^c^The lesser the distance, the tighter the composite grip

^d^Using the modified Kapandji score

^e^Quick Disability of the Arm, Shoulder & Hand Questionnaire

### Association of physical measures with QuickDASH scores over time

Table 3 shows results of the generalized least squares models of QuickDASH for the various ROM measures. None of the ROM-by time interactions were statistically significant, indicating that the associations between ROM and QuickDASH were consistent across time. Wrist extension ROM (8.95 [CI -4.94 to − 12.95] lower QuickDASH scores per IQR increase in wrist extension ROM), active thumb opposition (IQR-β, − 8.9 [− 2.73 to − 15.07]) and the ability to make a full composite grip (IQR-β, 7.64 [3.6 to 11.69]) were among the strongest ROM measures associated with QuickDASH scores. Other ROM measures that were statistically significantly associated with QuickDASH included wrist flexion (IQR-β, − 6.70 [− 11.50 to − 1.89]), forearm supination (IQR-β, − 6.57 [− 9.81 to − 3.33]) and wrist ulnar deviation (IQR-β, − 4.06 [− 7.21 to − 0.90]). In contrast, the associations of forearm pronation (IQR-β, − 0.63 [− 2.66 to − 3.93]) and wrist radial deviation (IQR-β, − 2.43 [1.28 to − 6.15]) were not statistically significantly associated with QuickDASH scores over time.

**Table 3:** Association of physical measures with QuickDASH scores over time^a^

Physical measures	Percentile^b^		Difference (95% CI)	*P*-value
	25^th^	75^th^		
Wrist flexion, degrees	25	45	−6.70 (−11.50, −1.89)	< 0.01
Wrist extension, degrees	35	55	−8.95 (−12.95, −4.94)	<.0001
Wrist radial deviation, degrees	10	20	−2.43 (−6.15, 1.28)	0.20
Wrist ulnar deviation, degrees	20	30	−4.06 (−7.21, −0.90)	0.01
Forearm supination, degrees	65	85	−6.57 (−9.81, −3.33)	< 0.001
Forearm pronation, degrees	65	85	−0.63 (−3.93, −2.66)	0.71
Distance to distal palmar crease (DPC)^c^, cm	0	2.5	7.64 (3.60, 11.69)	< 0.001
Active thumb opposition^d^	4	8	−8.90 (− 15.07, − 2.73)	< 0.01

^a^Results shown are from separate multivariable generalized least-squares models for QuickDASH scores during follow-up, adjusted for age, sex, edema, hand dominance, time since first therapy session, time since fracture, and treatment center. None of the interactions between the ROM measures and time (weeks since first therapy session) were statistically significant

^b^Adjusted differences in QuickDASH scores reflect a comparison between the 75th vs. the 25th percentile values of each physical measure. For example, with all covariates kept equal, patients with wrist extension at the 75th percentile (55 degrees) would have, on average, 9.0 points (95% confidence interval [95% CI] 4.9 to 13.0 points) lower QuickDASH scores than patients with wrist extension at the 25th percentile (35 degrees). This scaling is done to facilitate the interpretation and comparison of effect sizes of various ROM measures that are measured on different units

^c^The lesser the distance, the tighter the composite grip

^d^Using the modified Kapandji score

## Discussion

In a sample of 138 patients with non-complicated, conservatively managed distal radius fracture, we examined the longitudinal associations of 8 forearm, wrist and hand ROM measures with QuickDASH scores. Adjusting for covariates, we found that wrist extension, active thumb opposition (using modified Kapandji scores) and the ability to make a full composite grip (measured by the distance of finger tips to distal palmar crease) were among the strongest ROM measures associated with QuickDASH scores. In contrast, forearm pronation and wrist radial deviation were weakly and non-significantly associated with QuickDASH scores. To our knowledge, this study is the first comprehensive evaluation of the ROM measures associated with changes in self-reported functional recovery in a relatively large cohort of patients with DRF.

### Ability to make a full composite grip and thumb opposition

Previous studies evaluating outcomes of distal radius fractures were often limited to the examination of wrist and forearm ROM measures, as well as functional outcomes such as DASH or the Patient-Rated Wrist Evaluation (PRWE) [7–9, 22–24]. Little is known about the impact of hand ROM measures following a distal radius fracture [25]. In a study that evaluated 260 patients following distal radius fractures, it was found that the development of hand stiffness was associated with poorer functional outcomes [25]. Stiffness was defined as fingertip to DPC distance greater than 1 cm for any one finger and functional outcome was indexed by the DASH questionnaire [25]. Similarly, our study found strong associations between hand-related ROM measures – such as the ability to make a full composite grip and thumb opposition – and physical function over time. It is possible that the amount of edema in the hand may have contributed to both limited hand mobility and poor physical function but our findings persisted after adjustment for edema and other potential confounders in the multivariable analyses (Table 3). As hand function is often altered due to the involvement of the wrist joint post DRF, it may be overlooked frequently in post DRF rehabilitation, where the focus is usually targeted specifically at wrist ROM. Our study highlights the imperative need to maintain finger and thumb ROM during immobilization due to the detrimental effects on function when ROM is lost. Hence, occupational therapists need to intervene and educate patients with DRF during the immobilization phases to optimize function post DRF.

### Wrist and forearm ROM measures

In our study, we found that wrist flexion, wrist extension, ulnar deviation, and forearm supination were statistically significantly associated with QuickDASH scores over time. Specifically, on average, an IQR increase in these ROM measures was associated with 4.1 to 9.0 points lower QuickDASH scores (Table 3). Using a different outcome measure such as the QuickDASH, our results expand on previous findings [26] indicating that wrist extension and ulnar deviation ROM were correlates of functional activities. Our interpretation of these findings is that wrist extension and ulnar deviation form part of the dart thrower’s motion, which describes a direction of oblique wrist motion from radial extension to ulnar flexion [27]. As the dart thrower’s motion is highly functional and is required in a variety of occupational, household, and sporting activities [28–30], our results are not surprising.

On the other hand, our results appear to contrast with those reported by Karnezis & Fragkiadakis (2002), who found that neither the wrist flexion-extension arc nor the forearm supination-pronation arc movements were significantly associated with functional scores [31]. However, it may be difficult to compare our results with those of Karnezis & Fragkiadakis (2002) due to differences in study population (unstable versus stable distal radius fractures), study design (cross-sectional versus longitudinal), outcome measures (Patient-Rated Wrist Evaluation versus QuickDASH) and sample sizes (31 versus 138). More research is needed to elucidate the reasons for the discrepant findings.

### Wrist radial deviation and forearm pronation

In our study, forearm pronation and wrist radial deviation were weakly associated with QuickDASH scores, and these findings were not reported in previous studies. Nevertheless, to explain this null finding, we reason that limited forearm pronation may be compensated by elbow and shoulder movements during daily activities. Supporting this, Sardelli and colleagues (2011) reported a maximal forearm pronation range of 65 degrees required for typing on a keyboard which was still lesser than the maximal amount of supination (77 degrees) required in opening a door [32].

### Study limitations

Our study has limitations. First, the retrospective nature of our study implies that we were unable to standardize (i) the timing of referral from injury to the first occupational therapy session and (ii) the time intervals for the follow-up assessments (Table 1). Although we have adjusted our results for these time variables using continuous-time regression models, we acknowledge that the ideal study would have standardized these variables to optimize the internal validity of the results. Second, to facilitate data collection, we have chosen a self-report measure of physical function (QuickDASH). However, given the poor concordance between self-reported measures versus actual performance of functions [31, 33], we acknowledge that our findings could be strengthened and complemented by examining performance-based measures of ADL tasks such as the Jebsen Test of Hand Function.

## Conclusion

To summarize, in 138 patients with conservatively managed DRFs, we found that the various ROM measures have differential influence on upper limb physical function, with wrist extension, active thumb opposition and ability to make a full composite grip amongst the strongest ROM measures associated with QuickDASH over time. The results of this study have potential implications on efforts in providing treatment and outcome assessments amongst people with DRFs. They also highlight the need for intervention and education of patients with DRFs to maintain hand-related ROM during the immobilization phase, to optimize function post DRF. That said, well-designed randomized intervention studies are needed to confirm these association findings, and future studies should explore how ROM measures interplay with muscle strength and pain levels to influence function post DRF.

## Abbreviations

ADLs: Activities of daily living
CRPS: Complex regional pain syndrome
DASH: Disabilities of the Arm, Shoulder and Hand
DPC: Distal palmar crease
DRF: Distal radius fracture
IQR: Interquartile range
PRWE: Patient-Rated Wrist Evaluation
QuickDASH: Quick Disabilities of the Arm, Shoulder and Hand
ROM: Range of motion
SD: Standard deviation
